# Introduction to Creating Feminist Futures: Research Methodologies for New Times

**DOI:** 10.1080/08164649.2024.2373931

**Published:** 2024-07-10

**Authors:** Rebecca Coleman, Kat Jungnickel

**Affiliations:** a School of Sociology, Politics and International Studies, University of Bristol, Bristol, UK; b Bristol Digital Futures Institute, University of Bristol, Bristol, UK; c Sociology Department, Goldsmiths, University of London, London, UK

**Keywords:** Feminist, futures, methodologies, methods, creative practice, creative research

## Abstract

What role do and might feminist methodologies, with their prioritisation of ethical and political questions and interventions, have in creating futures? What kinds of futures are needed? What kinds of feminist imaginations should be cultivated, and how? What world-making practices might feminism (further) develop and/or invent? In the context of war, climate breakdown, pandemics, the resurgence of far-right politics, political upheaval and poverty, this special issue examines the role of feminist methods in creating futures that are desirable and necessary. This introduction to the special issue argues that feminism is especially well-equipped to examine and build new futures and that imagining and making different worlds can be helpfully understood as methods. We sketch out four key themes that we see as significant within the wide, varied and growing literatures on feminist futures and that are particularly important for the contributions gathered together here: *non-linearity; interruption and refusal; world-making and speculation; collaboration*.

Visionary feminism offers us hope for the future. By emphasising an ethics of mutuality and interdependency feminist thinking offers us a way to end domination while simultaneously changing the impact of inequity. (hooks 2015, 117)

Re-imagining worlds and re-making futures have always been central to feminism and its intersections with other critical, minoritarian movements, including queer, Black and anti-racist, disability and class-based practices. As bell hooks notes, feminism in its visionary forms can cultivate thinking and practices to shape more ethical and equal future worlds. In this special issue of *Australian Feminist Studies*, we take such a proposition seriously and make two wagers. The first is that, at a historical moment of war, climate breakdown, pandemics, the resurgence of far-right politics, political upheaval and poverty, hopeful approaches and visions of alternative futures are needed more than ever. In her introduction to a special issue of this journal on ‘Feminist Generations’, Rosi Braidotti notes that feminism has ‘a long and rich genealogy in terms of pleading for increased visionary insight’, which ties together ‘a strong critical and an equally strong creative function’ (2009, 8). In particularly turbulent times such as these, there is an urgency to revisit and perhaps revise the critical and creative approaches and practices through which different futures can be imagined and made. Here, then, we want to centre *futures* as an object and/or orientation that feminism is especially well-equipped to examine and build.

Our second wager is that, in considering feminist imaginings and makings of future worlds, it is productive to examine questions of *methodologies and methods*. That is, we think that imagining and making can be helpfully understood as methods through which different worlds can be devised. We take a deliberately broad understanding of methods, not only so that ‘method’ encompasses what can be the intangibility of imagination along with more practical making activities, but also to account for the variety of approaches that feminism has and continues to work with and through. These include practices that are core to specific academic disciplines, such as drawing and interviews, as well as those that cross and blur disciplines, such as reading and workshopping. They also include practices that take place outside of academic contexts, such as on websites, gaming, in classrooms and in activism. In this sense, we contribute to the recent resurgence of interest in methods as interdisciplinary (Lury 2018), creative (Hawkins 2013), material and embodied (Coleman, Page, and Palmer 2019; Jungnickel 2018), inventive (Lury and Wakeford 2012) and live (Back and Puwar 2012).

Against this background, we called for contributions to this special issue to respond to questions including: What role do and might feminist methodologies, with their prioritisation of ethical and political questions and interventions, have in creating futures? What kinds of futures are needed? What kinds of feminist imaginations should be cultivated, and how? What world-making practices might feminism (further) develop and/or invent? All of the contributions grapple significantly and in different ways with these questions, as we sketch out below. We begin by developing our second wager that methods – broadly understood – are central to critical and creative feminist thought and practice. We do this to situate broader feminist arguments about futures in methods, so as to carve out what is distinctive about the contributions we seek to make here. In particular, we identify four key themes that we see across different feminist research on futures: non-linearity; interruption and refusal; world-making and speculation; and collaboration. The final section introduces the articles and creative responses included in this special issue, pointing to how they take up and examine some of these themes and questions, and the approaches they set out and put into play.

## Feminist Methods

If, as Braidotti argues, feminism has equally strong critical and creative functions, nowhere is this more the case than in feminist methodologies and methods. We argue that methodologies and methods are crucial sites through which feminist imagination and making take place and new futures are envisioned and made. According to Yasmin Gunaratnam and Carrie Hamilton, feminist methodologies emerged as a field of academic interest in the 1980s. ‘Early discussions’, Gunaratnam and Hamilton argue,

suggested that feminist research and knowledge-making demanded a distinct approach to empirical inquiry: one that recognised and overturned systemic gender disparities, validated women’s “experience”, rejected hierarchies between the researchers and research participant, and had emancipation and social change as its purpose. (2017, 1)

Ongoing debate, contestation and experimentation with such ideas notwithstanding, ‘a commitment to make feminism mean something in the doing of research, cultural analysis, teaching, artistic practice and in activism, has continued to complicate and supplement the idea of a distinct feminist methodological imperative’ (Gunaratnam and Hamilton 2017, 1).

A number of points raised by this argument are significant. The first is that what constitutes feminist methodology and method is not unified or fixed. Multiplicity and diversity define feminist methods. Another point is that feminist methods are rigorously theorised, and the work on feminist research has therefore refused any straightforward distinctions between theory and practice; indeed, work on feminist research might be understood to compose and sit within a space whereby theory is practised and practice is theorised. The implications of this point are that methods are not tools, and that it is not possible, or desirable, to offer standardised versions or blueprints of how feminist methods should be put to use. Rather, the relationship between theory, methods and practice is dynamic: relational, changing and specific to the problems at stake (Lury 2020; Lury and Wakeford 2012).

A further related point is that, if methods are not instrumental – that is, if we do not see methods as tools that reveal or mine pre-existing realities – methods are world-making. Methods make worlds. This is what is apparent in Gunaratnam and Hamilton’s emphasis on the enduring ‘commitment to make feminism mean something in the *doing*’ (our emphasis). Karen Barad (1998, 2007), for example, writing about the instruments through which we know phenomena, argues that our ways of knowing are always entangled with the phenomena being researched: so that ‘tools’ are productive of, rather than reflections of or uncovering, that phenomena. As she argues, ‘practices of knowledge are specific material engagements that participate in (re)configuring the world. Which practices we enact matter – in both senses of the word. Making knowledge is not simply about making facts but about making worlds’ (Barad 2007, 91; see also Haraway 2016).

The performativity of what we are calling feminist methods – the co-constitutive entanglement of theory and practice, the doing of research, analysis, teaching, art, activism …  – requires us to ask political and ethical questions about the worlds we are, and are not, making (see also Law and Urry 2004). Whether or not explicitly acknowledged, these questions are always concerned with futures: they are about the futures that are imaginable, desirable and necessary.

## Feminisms and Futures

That the relationship between feminism and futures is long-standing and close does not mean that it is coherent or unified. In exploring feminist approaches to futures, then, a range of responses – none wrong, but all varied – are notable. Sara Ahmed argues that feminism is, by definition, ‘a desire that the future should not simply repeat the past, given that feminism comes into being as a critique of, and resistance to, the ways in which the world has already taken shape’ (2004, 183). However, this characterisation of feminism as inherently interested in futures emerges from a discussion of the contested grounds of feminism, especially around questions of the role of pasts in shaping futures, the inequalities that structure feminism and the differences that are part of any understanding and feeling of solidarity. ‘When we think the question of feminist futures’, Ahmed writes,

we also need to attend to the legacies of feminist pasts; what we have inherited from past feminists, in terms of ways of thinking the very question of what it would mean to have a world where feminism, as a politics of transformation, is no longer necessary. (2004, 183)

When we centre feminist futures, then, we are necessarily asking questions about pasts and presents. The future is not a time somehow segregated from what is and has been; rather futures, presents and pasts are always imbued with and configured through each other.

Also focusing on how futurity figures in and shapes feminism and the stories that are typically told about it, Clare Hemmings, writes about how she ‘always loved feminist theory for its utopianism’ and ‘dogged optimism that allows its practitioners to understand and experience life differently’ (2011, 3). Others zone in on the politics of utopianism and optimism. Lauren Berlant’s work on ‘cruel optimism’ (2011) details the requirement of critical theory to examine the attachments to ideals, hopes or fantasies of easier, better futures and how such attachments can defer action in the present. Hope and optimism can be regressive and conservative as well as transformational and revolutionary (Coleman and Ferreday 2010; Munoz 2009). One of the points that the debate about hope and optimism indicate is the role of feeling or affect in feminism. Caroline Bassett, Sarah Kember and Kate O’Riordan contend that feminism needs to galvanise a ‘furious’ response to ‘contest the regressively gendered and very often sexist politics of digital media forms, practices and study’, in order to ‘re-conceptualize digital media and broader technological futures, pervasive mediation and increasing automation’ (Bassett, Kember, and O’Riordan 2019, 2). Focusing on the contemporary terrain of social media, Jenny Sunden and Susanna Paasonen detail how feminist tactics on social media that use humour, laughter and the absurd are modes of ‘creative world-building’ that not only critique networked forms of sexism but also ‘disrupt[…] and eschew[…] the logic on offer’ (2020, 11). Laughter here is not so much coded as a ‘good’ or ‘bad’ affect or emotion as it is a potentiality that may ‘set in motion’ more or less enduring ‘social and political resonances’ (2020, 11).

There are clearly many issues that feminism has and continues to explore in relation to an understanding of futures, why and how they matter, and for whom. Our discussion here is focused on four themes that we identify as significant within the wide and growing literatures on feminist futures and that are particularly important for the contributions gathered together here: *non-linearity; interruption and refusal; world-making and speculation; collaboration*.

One tendency within feminist conceptualisations of futures, and that animate some of the special issue articles, is what Braidotti calls ‘[t]he scrambling of feminist time-lines today’ (2009, 6); that is, the problematisation of temporal linearity that emerges out of feminist philosophical work on women’s time as circular and cyclical and in attempts to understand contemporary capitalism and globalisation, in which time is organised around a continuous present/presence. For some feminist theorists, such temporal scrambling can be understood as a mode of self- or internal-reflexivity whereby the future of feminism is not secure, as Lisa Adkins (2004) argues; a non-dialectical, non-linear, transversal ‘jumping’ across and between feminist knowledge produced within different historical moments and theoretical traditions as Iris van der Tuin (2009) puts it; or a re-turning, or turning over, of persistent issues or problems so that, in Christina Hughes and Celia Lury’s terms, ‘there is no singular or unified progressive history or approach to discover. Rather, there is the intensity of multi-dimensional trajectories’ (2013, 787). While futures are not always explicitly at stake in these analyses, what such different versions of feminist timelines indicate is that feminist futures are neither pre-determined nor singular. And neither are feminist futures only to be distinguished by looking ‘forward’ or through empty versions of ‘the new’.

This point – that *futures are non-linear* – is carefully explicated in recent Black and queer feminist work. In her analysis of Afrofuturist cinema, music and literature, Kara Keeling examines the colonisation of the future through the corporate future scenario industry, and compares this to an interdisciplinary and collaborative imagination, which creatively and speculatively animates possibilities. Importantly for Keeling, while such future possibilities are ‘not-yet’, they are to be found in modes of queer temporality that ‘stubbornly persists in present relations’ and at the same time remains imperceptible in dominant ‘efforts to anchor the future to the knowable’ (2019, 20). In other words, Black futures are animated in and as the present (see also Nyong’o 2018). From a different, although complementary, position, Tina Campt ‘listens’ to photographs that document colonialism and its resistance, and argues that they anticipate and perform a ‘tense relationship to an idea of possibility that is neither innocent nor naïve’ (2017, 17). This is:

a grammar of possibility that moves beyond a simple definition of the future tense as *what will be* in the future. It moves beyond the future perfect tense of *that which will have happened* prior to a reference point in the future. It strives for the tense of possibility that grammarians refer to as the future real conditional or *that which will have had to happen*. The grammar of black feminist futurity is a performance of a future that hasn’t yet happened but must. It is an attachment to a belief in what should be true, which impels us to realise that aspiration. It is the power to imagine beyond current fact and to envision that which is not, but must be. It’s a politics of pre-figuration that involves living the future *now* – as imperative rather than subjunctive – as a striving for the future you want to see, right now, in the present. (Campt 2017, 17)

Both Keeling and Campt locate the future within the present; the future may be that which is happening now and yet is that which is somehow distinct from, better than, the present. Analysing how sociology traditionally approaches Black life as a ‘problem’, Nadine Ehlers argues that the discipline is stuck in ‘a recursive trap of simply naming these repetitions. Under this model, the only way to imagine or arc towards another future is to try to get *outside* the loop of imperilled black futurity’ (2023, 339). In contrast, Ehlers finds alternative presents and futures being made in grassroots black community initiatives, such as those which developed during the early phase of the COVID-19 pandemic in the USA and Australia. These ‘practices or tactics or living within the contours of imperilled black biofutures’, ‘push back against the recursive story to *rework* the enduring logics of anti-blackness’ by ‘fostering life against the various forms of letting (or making) die that are exacerbated in our current pandemic moment’ (2023, 341). Ehlers’ argument demonstrates that futures are ‘that which [are] in the making all around us’ (2023, 3423; see also Halford and Southerton 2023) and that ‘[w]e need to be attentive to and learn from *the alternative futures-now that are already here* and use these other futures-now to organise tomorrow’ (2023, 343). Cultivating methods that are attentive to and can learn from ‘futures-now’ is thus a central aim of any project interested in futures. Feminist theories and practices, we argue, provide us with a rich, powerful and plural range of resources to do this.

Another key thread weaving through these literatures and articles that follow is that of *interruption*. Feminist futures, much like feminist presents and pasts, are rarely smooth, streamlined or singular. They are complex, messy and knotted with overlaps and intersections of multi-dimensional experiences, knowledges, people, and things that don’t all necessarily perform or fit as expected. Legacy Russell writes about the glitch as ‘an error, a mistake, a failure to function’ (2020, 20). In some contexts, a glitch indicates interruption, a breakdown or problem, but it can be something else, more powerful and generative. The glitch ‘creates a fissure within which new possibilities of being and becoming manifest’ (2020, 11) which can ‘guide us through wayward worlds toward new frameworks and new visions of fantastic futures’ (2020, 14). A feminist glitch can be a ‘body that pushes back at the applications of pronouns, or remains indecipherable within a binary assignment’ or simply refuses to perform (2020, 21).

*Refusal* is another form of interruption in feminist, queer, disability, indigenous and decolonial scholarship. Ahmed writes about how feminism ‘can be understood as a trans/formative politics in its very refusal to belong to either here or there’ (1998, 43). Disability theorist Rosemarie Garland-Thomson describes ‘misfitting’ as ‘an incongruent relationship between two things: a square peg in a round hole’ (2011, 592–593). She powerfully draws attention to spaces which do not fit all bodies, and reverses the reading of this incongruity. Instead of assuming the disabled subject or ‘square peg’ is the problem, she argues that it is the ‘round hole’ or environment that does not work. Her work draws attention to contexts and systems that refuse access to some people and those who do not fit these homogenised norms for their ‘adaptability, resourcefulness and subjugated knowledge’ which emerge as ‘potential effects of misfitting’ (2011, 592). Collectively, these ideas carve out space, time and opportunity to re-imagine and re-make futures.

A further theme in feminist research on futures that is important for the special issue contributions focuses specifically on *world-making as a speculative project*. This theme is perhaps most explicitly inspired by Donna Haraway’s work on Sf

that potent material semiotic sign for the riches of speculative fabulation, speculative feminism, science fiction, science fact, science fantasy – and, I suggest, strong figures. In looping threads and relays of patterning, this sf practice is a model for worlding. Sf must also mean “so far”, opening up what is yet-to-come in protean times pasts, presents and futures. (Haraway 2013, 4)

Here, ‘speculative’ refers to experiments with the not-yet, which range from the curation of occasions through which new futures may take shape to what Lisa Adkins calls, 'the adoption of alternative stances towards sociological data, that is to recordings of social life' (2017, 117). What characterises the diversity of speculative research is a commitment to, in Vivienne Kuh's words, ‘“trouble [the] ontological entrapments” […] researchers find themselves in, but also initially, even to expose them in the first place' (2021, 272).

For Kuh, ‘researchers’ include those in research roles (academic, industry and otherwise) and those whom research touches, directly or not – ‘given the lack of inclusion across all our institutions, and the mounting costs of non-inclusive futuring, it is imperative’, she argues, for inclusion to be at the heart of speculative world-making. This is not the additive version of inclusion that feminism has challenged for so long, but rather is akin to what Sasha Constanza-Chock (2020) calls ‘design justice’: practices led by marginalised peoples whereby many worlds are imagined, shaped and built (see also Escobar 2018).

Our final theme is *collaboration* – evident in the many co-authored papers included in the special issue. Few feminist projects are solo endeavours. Even when they are not deliberately co-constructed and enacted, they are deeply situated within or in relation to frameworks or networks. For some, collaboration is *always* a feminist practice. In defined pairs or groups, feminist collaborations can transform traditional power dimensions, subverting normative hierarchies and lead to long-term care, support and inspiration. Reflecting on such issues in terms of their long-term collaboration, Carey Kaplan and Ellen Cronan Rose write, ‘collaboration among academic feminists, conducted as it is against institutional odds, is exhilarating, consoling, and precious’ (1993, 559) – which is not to say easy or straightforward. It is often under-appreciated, under-explored and under-funded. It is also hard to sustain, given the academy’s predilection for solo-authored publications and endeavours (see also Gilbert and Masucci 2008). Discussing how they have embedded feminist principles of care, process and equity into the feminist marine science laboratory she leads, Max Liboiron and colleagues have explored different ways in which collaboration can be made explicit, including through collectively coming to a consensus about author order (Liboiron et al. 2017) and shifting extractive reading and writing processes into more inclusive practices such as inviting readers to participate as collaborative co-authors (Liboiron 2018). Highlighting the importance of collaboration to this special issue – through co-writing, participatory methods and approaches and also through broader programmes of research – helps to demonstrate its potential for creating feminist futures.

## Contributions to the Special Issue

The themes we outline above criss-cross through the contributions to the special issue, which span academic articles, conversations about and reflections on feminist research and creative practice pieces. They locate and examine feminist futures methodologies in relation to the doing and analysis of empirical social research and art and design practices, and also in classrooms and kitchens, on websites, Zoom calls and mobile games, through friction and fiction, and multispecies flourishing.

Esther Priyadharshini considers her empirical futures-making project with young people in the face of ‘wicked problems’ such as the climate crisis, poverty, far-right politics, poverty and the COVID-19 pandemic. Designed as a speculative research project, it built on Afrofuturism, in which publics are invited to participate in ‘conjuring and enjoying new sensibilities of how a multiplicity of futures may break away from tedious, singular teleological frames’ and Black feminist science, in which ‘disobedient, rebellious and rogue’ ‘method-making’ makes new worlds. Priyadharshini carefully considers the practicalities of the research project, and ‘fleshes out’ a methodological framework to ‘bridge the world of empirical research with its particular challenges, and the inspirational-aspirational world of Black Feminist Science and Afrofuturism’. This methodological framework functions as a series of ethical, political, methodological and practical questions that prompt interested researchers to focus attention on how research may aid in creating feminist futures.

Exploring their empirical research in Finnish secondary schools and UK Further Education to re-imagine what more fulfilling classrooms and lessons might be, Katja Hiltunen and Greg Campbell devise methods of speculative fabulation inspired by the different feminist new materialisms of Barad, Braidotti, Jane Bennett (2010) and Haraway. They riff off of their ethnographic observations to tell different stories and write poems about more diverse, inclusive and enjoyable classroom experiences, showing both how such futures are illuminated in the present and the potential of asking ‘what if … ?’ questions in allowing new futures to take shape. These methods world-make differently in small ways and have the potential to have more major affects and effects.

Also considering the methodologies via which feminist research may be conducted and feminist and queer futures made, Lindsay Kelly tracks the development of her participatory taste workshops, in which people bake and eat Anzac biscuits, as they moved from being hosted ‘in real life’ to online spaces during the COVID-19 pandemic. She argues that the requirement to move to Zoom workshops opened up her understanding of the normative assumptions that ‘being together in-person’ is based on – both in terms of COVID-19 lockdowns and for participatory methods more widely. Exploring the online workshops in relation to digital artworks, she argues that they ‘reveal how togetherness must be continually made up and created as feminist, queer affordances’ and ‘trigger debates about how collectivist and feminist futures should operate moving forward’.

Kiera Obbard and Lauren McLead take an understanding of methods away from academic research to Audostraddle, a digital community and publication for and run by LGBTQIA2s + people, which they argue functions to queer digital space and create feminist futures. Instability and tension are central to their analysis and object of study, not only in relation to the content and audience of the platform but also how it operates in the context of hegemonic power structures. They reflect on how ‘the long-term viability of these spaces is ultimately dependent on capitalist founding models’; which requires ‘finding ways to queer this model from within’. Yet, they found that while Autostraddle attempted to interrupt the system, it still remained tied to and constrained by it. They argue that ‘perhaps Autostraddle is able to truly evoke a queer framework *because* of the internet’s capitalistic limitations’. This doesn’t mean it has failed, and given up on its queer intent, but rather that in its refusal to conform it ‘adapted as a means of survival’. This flexibility might well make it stronger and more resilient in the long term.

Liu Xin reflects on the process of co-designing an experimental mobile game, Square Cat: Idle Fish Eater, in which a cat destroys and eats the entire earth. Xin explores the co-creation of the game as a feminist methodology of making, as well as analysing, futures. Contextualising Square Cat in wider debates about how games and gamification can help publics imagine futures, Xin argues that the feminist methodology of co-designing it ‘games the game’. Complicating binary ideas of futures as either opened up or foreclosed through games, or of futures as either good or bad, Square Cat dramatises the logics of gaming, including of winning and losing, and ‘ask[s] the player to entertain the possibility of playing a game […] where the future might be multiple even as the beginning and ending of the game seems to be provided in advance’.

In their contribution, Åså Ståhl, Saskia Gullstrand, Li Jönsson and Kristina Lindström also complicate dominant narratives of futures – what they call the ‘thin stories’ of dystopian and tech-utopian futures – through their co-creation design-led collaboration project, Un/Making Pollination. Attending to the decreasing number of pollinators and a lack of imagination about the possibility other worlds, the project created a series of posters on pollination as feminist methods of opening up ecosocial imaginaries. The paper presents a critical account of the ‘method assemblage’ of creating and sharing the posters, which draw from academic and activist work and highlight the importance of situatedness, reflexivity, complexity and ambiguity. Rather than provide ‘thin stories’, they argue the posters work through ‘the poetic artistic expression of clarity and smudginess’, luring viewers into close inspection and ‘invit[ing] an experimentation regarding how to understand thick entanglements’ through which presents and futures can be re-made.

Also examining questions of multi-species, environmental futures, in the Feminist Forum section, Ekaterina Gladkova and Naho Matsuda take up urgent issues of present and future food production and apply a feminist lens to the industrial farming of pigs. Drawing on a research project called Re:Pig, they argue that human and more-than-human entanglements have the potential to be shaped not just by exploitation and domination but by multispecies connection and kinship. This paper puts feminist methods into practice in the form of a visual essay featuring speculative scenarios from an illustrated zine. Each scenario sets out to provoke and ‘re-think the compromised lives of farmed pigs and other affected species and challenge the conceptions of farmed animals’ agency’.

Another take on how ‘staying with the trouble’ can elicit new futures is found in Jessamy Perriam, Marisa Leavitt Cohn, Michael Hockenhull, Lara Reime, Luis Landa, Katrine Meldgaard Kjær and Henriette Friis’s article about ‘workshopping troubles’. Written about and by members of ETHOS, the feminist STS lab at Copenhagen’s ITU that specialises in digital methods, the collective reflect together on workshops that aimed to reveal insights into their collective practice, but instead of ‘congealing a set of feminist principles’ they surfaced ‘proliferations of disconcertments and troubles’. Drawing on Haraway’s work, the lab understands these as requiring them to ‘stay with the trouble’; of representation, of the discomfort of researcher accountability, and with consent as never done. The lab therefore offers ‘workshopping troubles’ as ‘a method for navigating research design choices in digital methods projects that supports negotiating the world-making in which we participate’.

Eliza Chandler, Megan A. Johnson, Chelsea Jones, Elisabeth Harrison and Carla Rice pay close attention to their experiences of conducting research through critical access methodologies, in which ethical and political questions of dis/ability and access are prioritised. Reflecting carefully on different projects that mobilise citizen journalism and artistic research methods, they highlight how moments of friction – where their attempts to practise critical access methodologies through the conception, design and processes of their research – sensitised them not only to problems and failures but also to how friction may itself be transformational. These authors argue that ‘moves towards feminist crip futures are rarely utopic, but are full of frictions, occlusions, grapplings, and restarts’. Understand in this way, critical access methodologies can signal and prefigure ‘crip feminist futures’ ‘in which disability and difference are expected, welcomed, and positioned as vital and generative ways of being in the world’.

In the Feminist Forum section, Nithilia Kanagasabai also trains her eye on the frictions, fictions and fissions that characterised her research, which focuses on the figure of the Indian doctoral researcher engaged in producing feminist knowledge of India from a position in a US university. In particular, she examines her interactions with her interlocutors through screens, as she contacted, recruited and interviewed from afar them via email, social media, and Skype, and argues the screens underscore the partial, mediated nature of the ‘epistemic inequalities’ of uneven geographies and transnational research. Indeed, the screens highlight how interviewing may not, or not only, involve the emotional closeness that feminist work on methodologies has long argued for (e.g. Oakley 1981), but (also) frictions that reveal global power relations, fragments that trouble seamless, unified, holistic stories and failures that mark how specific types of knowledge production become universalised and hierarchised. Staying with such moments of friction and discomfort provide the possibilities to ‘reverse the ethnographic gaze […] in order to (re)imagine a more equitable feminist research future’.

Justine Grønbæk Pors and Signe Ravn contribute to feminist and queer research by developing a methodology through which to identify and articulate the alternative futures that they see as latent within empirical research. Focusing on their research projects on young women’s imagined futures, they trouble prevailing theoretical accounts of future-making as linear processes by analysing how their research data shows time and futures as non-linear. They do this through an affective practice of ‘reading against the grain’, in which disconcerting or intense moments, which might seem minor, can be amplified to ‘go beyond normative, chronological narratives’. The value of such a reading strategy, they argue, is to consider ‘methods’ beyond questions of research design so that methods of analysis – including of data generated through well-established methods such as interviews – can be seen as ‘shedding light on other ways of making futures and being in time than the linear norm’.

Finally, the conversation between Gökçe Günel, Chika Watanabe and ourselves explores the work of Patchwork Ethnography, launched by Günel, Watanabe and Saiba Varma in June 2020. Patchwork Ethnography seeks to re-think the method of ethnography in the context of changing organisational structures in the university, gendered work practices, caring responsibilities, the climate crisis and the pandemic, drawing attention to and seeing much value in how many researchers work with and around these challenges and pressures, stitching together relationships with participants, fieldwork and outputs. In the conversation, we collectively explore what patchwork ethnography involves, how it is informed by feminist work, why it hit a nerve with so many researchers, and what its futures might be. The conversation includes reflections on methodology and method, writing practices, personal and professional relationships, labour, mess and what ‘patchwork’ offers politically and ethically as a means of creatively ushering in other kinds of world-making.

Despite the differences in their research approaches, foci and presentation, across all of the contributions is a close attention to the ways in which radical social, cultural, economic, political and ecological shifts require urgent, ethical responses. As Arundhati Roy argued in the early days of the pandemic:

Nothing could be worse than a return to normality. Historically, pandemics have forced humans to break with the past and imagine their world anew. This one is no different. It is a portal, a gateway between one world and the next. We can choose to walk through it, dragging the carcasses of our prejudice and hatred, our avarice, our data banks and dead ideas, our dead rivers and smoky skies behind us. Or we can walk through lightly, with little luggage, ready to imagine another world. And ready to fight for it. (Roy 2020, np)

Feminist methods – capaciously defined and deployed – have a crucial role in this critical and creative re-imagination and re-making of the worlds we can and do want.
